# Reducing episodes of diabetic ketoacidosis within a youth population: a focus group study with patients and families

**DOI:** 10.1186/s13104-015-1358-7

**Published:** 2015-09-01

**Authors:** Roger Chafe, Daniel Albrechtsons, Donna Hagerty, Leigh Anne Newhook

**Affiliations:** Janeway Pediatric Research Unit, Division of Pediatrics, Faculty of Medicine, Memorial University of Newfoundland, Room 409, Janeway Hostel, 300 Prince Phillip Drive, St. John’s, NL A1B 3V6 Canada; Memorial University of Newfoundland, St. John’s, NL Canada; Eastern Health, Outreach Department, St. John’s, NL Canada; Division of Pediatrics, Faculty of Medicine, Janeway Child Health Care Centre, Memorial University of Newfoundland, St. John’s, NL Canada

**Keywords:** Type 1 diabetes, Diabetic ketoacidosis, Patient experience, Patient resources, Rural

## Abstract

**Background:**

Diabetic ketoacidosis (DKA) is the most common cause of morbidity and mortality for youth with type 1 diabetes mellitus (T1DM). This article reports qualitative data from focus groups with youth and parents of youth with T1DM on the barriers that they identify to DKA prevention and resources that may aid youth better manage their diabetes.

**Methods:**

Four focus groups were held in three communities, two rural and one urban, in the Canadian province of Newfoundland and Labrador (NL) with adolescents and parents of youth with diabetes. Open-ended questions focused on knowledge of DKA, diabetes education, personal experiences with DKA, barriers to diabetes self-management, situations which put them at risk for DKA and resources that could be developed to aid youth in preventing DKA.

**Results:**

There were 19 participants (14 parents and 5 youth). Participants identified factors which increased their risk of DKA as difficulty in distinguishing cases of DKA from other illnesses; variations in diabetes education received; information overload about their condition; the long period from initial diagnosis, when most education about the condition was received; and stress regarding situations where youth are not in the direct care of their parents. Participants from rural areas reported geographical isolation and lack of regular access to specialist health care personnel as additional barriers to better diabetes management.

**Conclusions:**

The project identified barriers to DKA prevention for youth which were not previously identified in the medical literature, e.g., the stress associated with temporary guardians, risk of information overload at initial diagnosis and the long period from initial diagnosis when most diabetes education is received. Families from rural areas do report additional burdens, but in some cases these families have developed community supports to help offset some of these problems. Mobile and online resources, educational refreshers about DKA, concise resources for teachers and other temporary guardians, and DKA treatment kits for parents may help improve diabetes management and prevent future episodes of DKA.

**Electronic supplementary material:**

The online version of this article (doi:10.1186/s13104-015-1358-7) contains supplementary material, which is available to authorized users.

## Background

Diabetic ketoacidosis (DKA) is a serious complication of diabetes, resulting from a severe lack of insulin and is the most common cause of morbidity and mortality in youth with type 1 diabetes mellitus (T1DM) [1, 2]. There are numerous factors that have been reported as causes of DKA, including problems with the patient’s insulin delivery system, mismanagement of insulin dosing during periods of illness or stress, and non-adherence with insulin therapy [1–4]. Yet most cases of DKA are preventable through proper insulin management and early detection of diabetes for new patients. Vanelli et al. and King et al. both report on successful public education programs for reducing incidences of DKA for patients who are first presenting with diabetes [5, 6]. For patients who know that they have diabetes, the importance of regular self-monitoring of blood glucose and ketones [7], ongoing education, telephone counseling, and early outpatient treatment [8] have be described.

Patients and their families play a crucial role in managing diabetes and have a major impact on outcomes, including preventing DKA. Families also have a unique perspective on the challenges they face on a daily basis and the supports that are likely to work in the real world to improve self-management. In order to help plan for a comprehensive prevention program for DKA, we invited patients and parents of patients to a series of focus groups to discuss their experience with T1DM and DKA. The primary objective of our study is to hear from youth (12–18) and parents of youth with T1DM about the factors that they perceive as putting them or their children at greater risk of having an episode of DKA and which resources they feel would be best to put in place to lower their risk. While DKA can happen at any age, we focused on youth with T1DM because of the unique issues that youth face as they take on increased responsibility for the management of their disease, [9, 10] the high rates of DKA within the youth population [11], and that it was in keeping with the focus of the larger research program. To the best of our knowledge, our study is the first qualitative study to engage youth and families directly on the topic of DKA and its prevention.

We conducted these focus groups with patients and families as part of a larger project focused on reducing rates of DKA in children and youth in the Canadian province of Newfoundland and Labrador (NL). NL has some of the highest recorded rates of T1DM in the world, with an average incidence rate from 2007 to 2010 of 49.9 cases per 100,000 per year (95 % CI 42.2, 57) [12, 13]. It has also been shown to have high rates of DKA hospitalizations within its pediatric and youth populations and the rate of DKA in newly diagnosed patients of only 22 % [10]. Some studies have indicated that rural areas display a higher incidence of type 1 diabetes [14], have issues with implementing education programs [15], lack of appropriately trained professionals to run prevention programs [16] and have less frequent visits with diabetes specialists [17, 18]. Because families in rural area may face different types of barriers, we conducted half of our focus groups in rural communities, which were defined as communities with less than 10,000 people and “outside the commuting zone of larger urban centres” [19].

## Methods

The inclusion criteria for the focus groups were either: (1) youth (age 12–18 years) with T1DM, or (2) a parent or primary guardian of a youth (age 12–18 years) with T1DM. Youth were not required to have had an episode of DKA. All focus groups were facilitated by one of the researchers (DA) using a semi-structured interview guide, with another researcher (RC) observing the first focus group. Only the participants and the facilitators were present during the focus group sessions. Focus group questions (“Appendix”) were developed by the authors based on the study aims and were reviewed multiple times by the project’s advisory committee, which included a teenage patient with T1DM, a parent of a youth with T1DM, two diabetes nurse educators, a pediatric endocrinologist and diabetologist, and other researchers. For the rural communities, focus group questions were also reviewed by local care providers, but were deemed as not needing to be changed from the questions used in the urban focus groups. Because the focus groups were all facilitated using a semi-structured interview guide, participants had the opportunity to ask for clarification of questions and to raise other issues that they thought were relevant to the project. For the first rural focus group, the diabetes educator contacted potential participants from the local diabetes clinic. For the first urban focus group and the second rural focus group, participants were recruited through invitations mailed out to all families attending their local diabetes clinic. For the second urban focus group, a local support group for parents of children with T1DM helped to recruit participants through an invitation sent out to their members. For all of the focus groups, the first eight people who responded and met the inclusion criteria were invited to attend the focus group session.

Focus group sessions were all recorded and professionally transcribed. Field notes were also taken for all sessions by the researcher who facilitated the focus groups. All of the transcripts and field notes were reviewed in their entirety by two authors (RC and DA) before coding to help determine the end of the data collection phase of the project. Based on the quality of the data collected and the repetition of themes, the team concluded that there was sufficient data not to warrant further data collection after the fourth focus group [20]. Two authors (RC and DA) then coded the transcripts independently using NVivo 10 software [21]. Because of the project’s focus on capturing perceived risk factors and potentially useful resources, we developed sub-codes for each unique risk factor or resources, reviewing each to ensure that none of the discussed risk factors or resources were missed. Key themes were discussed and clarified by three of the authors (RC, DA and LN) and reported to the project’s advisory committee. Written consent was obtained from all participants prior to the focus group sessions and written assent was obtained from minor participants. Ethics approval for the project was obtained from the Newfoundland and Labrador Health Research Ethics Authority [22].

## Results

There were 19 participants; 5 were youth with T1DM and the rest were parents of youth with T1DM, including the parents of the 5 youth who participated. Focus groups ranged in size from 2 to 6 participants. Four of the parents in the focus groups reported that their child had previously experienced DKA. The youth who attended the first urban focus group had two previous cases of DKA. One episode of DKA was due to an illness (gastroenteritis), which led the family to deviate from their regular diabetes management. The cause of the other episode was undetermined and happened while as a child and she was in the care of her grandmother. The unpleasantness of the experience of DKA and being treated in hospital for it was referred to throughout the focus group. In the second urban focus group, participants had experienced two cases which occurred at diagnosis of T1DM and another case related to another illness. None of the participants in the rural focus groups had experienced DKA. Additonal file 1: Table S1 details the location (i.e., whether urban or rural), date and compositions of the four focus groups as well as the gender of the youth participants and the number of cases of DKA discussed in the focus group and the cause identified for the resulting DKA. To help ensure confidentiality, the names of the communities in which the focus groups were held has been suppressed.

## Barriers

Within all four focus groups, participants identified a number of barriers to the management of their or their child’s diabetes and to the prevention of DKA. In this section, we discuss the main barriers identified.

### Difficulty identifying cases of DKA

A key barrier discussed in every focus group was that it is difficult to tell the difference between cases of DKA and other types of illness. (“My daughter was sick and it was just like stomach flu sick, and how do you tell the difference?”—a mother of child who had an episode of DKA, 2nd urban focus group.) (“Because high blood sugars can be when they are sick, it is normal to have high blood sugar. So how do you tell the difference?”—mother, 2nd urban focus group.) Some participants said that they only felt able to clearly recognize the symptoms of DKA after it happened to their child. (“Once they experience it for the first time… once they experience those symptoms themselves physically, then they can, they’re more aware of it”—father, 1st rural focus group.) The issue of difficulty in identifying cases of DKA also extended to the recognition that it is even difficult for trained medical staff to identify cases. (“She [my child] went to DKA in emergency. She wasn’t brought in because she was in DKA. But while she was there… so she was actually in the care of medical staff. I mean we had no idea, we never experienced it before… [we] assumed that the medical staff would have known that this process was happening”—mother, 2nd urban focus group.) But parents also said that concerns about recognizing cases of DKA may get easier as children get older and be less of an issue for youth. (“I think maybe with younger children it’s harder, but… in adolescent or teenagers, they kind of, they know what’s coming on and kinda of can tell people”—mother, 1st urban focus group.)

### Variation in diabetes education

Another key theme which was discussed in all of the focus groups was that there was a good deal of variation in the level of education families received about DKA at the time of diagnosis and after. For two participants, their child experienced DKA during the initial presentation of diabetes. These families did receive detailed information about DKA from their child’s health care team. One of the other participants who had experienced DKA said that they had not been well-informed about it or how to prevent it, or had forgotten this information, before their child experienced DKA. (“She was admitted and they brought us up a test kit and they brought us up needles and showed us how to do this and how to do that, but they never said anything about DKA, absolutely nothing”—mother, 1st urban focus group.) Other people said that they did receive a good deal of information about diabetes and DKA at first diagnosis. (“Initially when we first went into the [hospital] and through the week of training and stuff, any sugar over 14, we were advised to double check for ketones”—father, 2nd rural focus group.) Other participants did not feel that they received sufficient information. (“I didn’t get much information back then. My son is thirteen now and he was diagnosed when he was 21-months and all that I remember we went through the whole week of training and met with the dietician, the doctor and what have you and I just came out of there with a little tiny kit, few brochures and I just went home and googled everything”—mother, 2nd urban focus group.) ([The medical staff] “were so busy that I didn’t find that we got all the information” father, 2nd urban focus group.) Many participants who had never experienced DKA said that the only DKA specific education they had received was at their child’s initial diagnosis. Other participants said that the information they received about DKA was contained within “big old binder” (youth, 2nd rural focus group) filled with information on living with and managing T1DM or “some pamphlets” (father, 2nd rural focus group). Differences in the geographic location where people were initially diagnosed also appeared to play a role in the education received. (“I don’t know what the rate of DKA in the Ottawa was at that time… we went to clinic every 3 months just as we do here and, but I mean, still no, no discussion of DKA”—mother, 1st urban focus group.) Some said that it was not until they had face-to-face sessions with a diabetes educator or pediatrician on the topic, well after their initial diagnosis, that they retained information about DKA. None of the youth participants reported receiving any DKA-specific education since their initial diagnosis.

### Information overload at initial diagnosis

Most of the education that people receive about their or their child’s diabetes was delivered when they were first diagnosed. One issue identified numerous times was “information overload” during this period. Parents said that there is a great deal of information given initially about managing and living with diabetes and it is easy to overlook more specific complications of diabetes, such as DKA. (“When your child is first diagnosed they talk about DKA, but you really have no concept of that because you are so wrapped up in everything else”—mother, 2nd urban focus group.) (“It is information overload almost because it’s not that the information wasn’t… It’s not that I wasn’t told and I haven’t gone through it but I think that you are just so overwhelmed with everything”—mother, 2nd urban focus group.) (“It’s like information overload. You’re just trying to cope with the change”—mother, 2nd urban focus group.) Particularly in the context of just being diagnosed, it can be difficult to comprehend all of the different adverse outcomes. (“If you started thinking about everything that could go wrong with them [your child], you would go crazy”—mother, 2nd urban focus group.) Another issue related to the way families are educated about the condition is that parents felt that the bulk of the education occurs when a child is first diagnosed. In many cases, the child is too young to benefit for much of this initial education.

### Caregivers other than parents

Situations in which the youth is not in the care of their regular guardian were often identified as times when youth are particularly at risk for both severe high or low blood sugar levels. Although only one youth reported actually experiencing DKA while outside of parental care (i.e., when with their grandparents), several others had episodes of very high blood sugars while not with other family members. Of particular importance for participants in every focus group were times when the youth was in school. The challenge of educating teachers about diabetes was a concerned which often rose, especially for youth in middle or high school, as students may have multiple teachers during the day. (“I worry about her in school because a lot of the teachers don’t know” [my child has diabetes]—mother 1st urban focus group; “where my school is so big, we have so many teachers and most of them don’t know [I have diabetes]”—youth 1st urban focus group.) Some of the parents found it difficult to go personally into the school and explain diabetes management to all of their youth’s teachers. (“I think that the Guidance Counselors in the schools need to have a solid education on diabetes for sure, because it doesn’t happen. I mean, like this year’s difficult because she does have 8 different teachers, so I mean, I can’t go to every single teacher”—mother, 1st urban focus group.) (“A huge need. … it’s the parents who are left to educating the teachers”—father 2nd rural focus group.) In most schools in the province of Newfoundland, the school’s guidance counselor would address health issues within the school, supported by a public health nurse who visits only periodically. Both youth in the second rural focus group had at one point been the only child in their school with T1DM, which added to their sense of burden. (“I know initially when (youth’s name) was diagnosed (youth’s name) was the only one in the school with diabetes”—father 2nd rural focus group.) Their parents felt that they had “to take the time to talk to all the teachers about my child” to ensure that their child would be properly cared for when they were not there (mother, 2nd rural focus group). While recognizing that temporary guardians can be a concern, participants also reported that a number of teachers are very good with allowing children extra time to manage their diabetes and identifying when a child may need some assistance. (“I must say, the teachers are good”—youth 2nd rural Focus group.)

### Sense of crisis

Issues were raised about a sense of crisis occurring when parents recognize that their child is or may be experiencing DKA. (“I was just in panic mode”—mother whose child experienced DKA, 2nd urban focus group.) Although it did not affect the ultimate outcome for the child, the added stress for parents can perhaps be seen as an additional adverse outcome of DKA. Similarly, some parents felt it was partly their fault that the episode occurred. This stress even caused guilt about the episode. (“So it was part my fault the first time because I didn’t understand. You know, I think of myself as being very informed on Type I diabetes, I’ve lived with it for 13 years, and to miss it, was kind of like, we need to get more information on this.”—mother, 1st urban focus group.)

### Rural issues

Even the participants from the urban focus group recognized the added burdens associated with living in rural areas with diabetes. (“I pity anyone from out of town somewhere that comes in and goes through all this training and got to go home”–mother, 2nd urban focus group.) Participants in rural areas reported having a greatly reduced access to specialist care was and a lack of immediate access to supplies. (“We need resources. That’s a, that’s a barrier. We need resources”—mother, 1st rural focus group.) (“We have a part time Dietician who spent one hour with us. You know, that’s, that’s not enough. Our child deserves to have an entire team. … a Social Worker. A Psychiatrist. A full time Dietician”—mother, 1st rural focus group). Being isolated geographically played a role in some parents’ attitudes towards allowing their children to travel on school trips for example. (“A big worry is when the child is outside the circle of care”—father, 2nd rural focus group.) In the first rural focus group, there were a number of participants whose children had experienced high blood sugars while the youth was outside the direct care of their parent. In one case, the teen had extremely high blood sugar while visiting a sports competition over 1000 miles away from their home. This experience was extremely difficult as the parent was unable to book a flight or drive to be with their teen who was admitted to hospital. (“I call over here to the airport—both flights are booked the next morning, so I didn’t even know if I could fly out if I was in a state of an Emergency—mother, 1st rural focus group). This parent said that while she used to be more concerned with low blood sugars, she is now very frightened of high blood sugars.

While both rural focus groups expressed that geographical isolation and a lack of resources were barriers to ideal diabetes management, all of the participants had used community supports to help them partially overcome these obstacles. (“I found we always were so used to doing everything ourselves from day 1”—mother, 2nd rural focus.) In the first rural focus group, the families all were regular attendees at a local support group for parents of children with diabetes. (“The support [group] is huge… because you do feel like you are alone [without it]”—mother, 1st rural focus group.) The second rural focus group also expressed that the social support and community ties offered by living in a smaller community aided their children. These stories indicate some possible advantages for people with diabetes living in a rural area.

### Suggested resources

Participants in all of the focus groups recommended that more resources are needed for temporary guardians, most notably teachers. One parent recommended that children be encouraged to use diabetes as subject material for speeches and presentations required in school. Through these activities, a child learns more about their own condition, but also educates others around them about what it is like to live with the disease. Friends in particular were seen as an excellent support for youth. (“One of the children that were hanging around with my son at the time [when her son’s sugars were very high] called his mom, luckily… so she knows our cell phone number so she kept calling us. They took it serious.” mother, 1st rural focus group.) (“My friends remind me to take my carbs, take my sugars”—youth, 2nd rural focus group.) One parent suggested that having a designated friend in school who knows about the youth’s diabetes is helpful for communicating with teachers, particularly in emergency situations. It is important also that these resources allow for teachers to be informed, but allow some degree of confidentiality for the youth with T1DM. (“Okay, this is what I have to do in case something’s wrong, look around the room, okay, these are the kids… I, you know, I got it in my mind what I need to do if something happens, not to have them posted up on the wall”—father, 1st rural focus group.) ([It needs to be confidential] “Cause they [youth with diabetes] want to feel like everyone else” mother, 1st rural focus group.) Small, laminated cards with clear directions for managing high and low blood sugar which can be given to teachers or temporary guardians were suggested.

Participants made a number of suggestions regarding the need for resources specifically made for children and youth with T1DM. (“[The education content] needs to be geared towards the kids… it needs to be something that the kids can relate to without the parents” mother, 2nd urban rural group.) Other parents thought that peer mentoring and resources created by the patients themselves would be helpful. One parent mentioned that their child had made his own “educational videos” which he has shown to friends and teachers. Another parent recalled how their child was deeply affected by the experience of watching older youth speak about their life with diabetes at a local diabetes event. (“Seeing other kids talk about their life with diabetes was really inspiring for him” mother, 2nd urban focus group.) Several parents touched on the themes of using the internet as a resource, specifically for short videos to educate patients, families and other caregivers. Combining a number of these themes, one parent said that “it would be great if you made a website using ideas and content submitted by the kids” father, 1st rural focus group.)

As part of the larger DKA prevention project, members of the research team (LA and DH) developed a DKA prevention kit, which had been distributed to families of children and youth with T1DM. These kits contain urinary ketone strips, insulin, needles, simple instructions about what to do if blood sugars and or ketones are high, as well as 24-h contact information for local health care providers. These DKA prevention kits were mentioned in three of the four focus groups as a good resource for parents. (“The kit is very helpful, very helpful. It’s right at your fingertips if we go anywhere. If we’re going on an extended vacation or going somewhere, I always have it stuck in a bag” mother, 1st urban focus group.)

## Discussion

The overall goal of our project is to reduce the rates of pediatric DKA. In this phase of the project, we examined the barriers to self-management of diabetes and determined what are the most appropriate supports needed by families to help reduce episodes of DKA. There are a number of factors which can result in children with type 1 diabetes developing DKA [2]. The most common causes of DKA may change as the child matures or if their diabetes management changes, for example switching from insulin by injection to insulin pump therapy. This fact can place increased importance of having good patient education as part of the patient’s transition into adolescence and then adult care. Participants identified factors which increased their risk of DKA as difficulty in distinguishing cases of DKA from other illnesses; variations in diabetes education received; information overload about their condition; the long period from initial diagnosis, when most education about the condition was received; and stress regarding situations where youth are not in the direct care of their parents. While the study area does have access to 24 hours a day diabetes crisis telephone line, still families often feel like they are on their own when dealing with these issues. The fact that the issues which these patients are identifying are occurring within the context of having access to efficient diabetes health care team, should make providers consider the importance to communicating with their patients, even around issues they may assume that patients know.

We intentionally focused on capturing the experience of families in rural areas. Proper self-management of diabetes is likely especially important in rural areas, where people can have decreased access to health care services, including diabetes specialists [23]. Some issues were reported relating to the low number of youth with diabetes in some of the communities, which placed added burdens on the parents for educating teachers about their child’s condition. But there were also advantages to living in rural communities, related to its size and connectedness. In one of the rural communities, parents have formed an active support group to help compensate for their increased isolation. Participants in the other rural focus group we conducted talked about the willingness of teachers to learn about their child’s condition.

To summarize the main recommendations from families and youth regarding their needs related to DKA education and prevention include:

1. DKA is a very stressful and traumatic event for youth and their parents.
2. Parents receive too much information at the time of diagnosis and DKA teaching is not retained. There tends to be a long period between teaching at initial diagnosis and re-education on complications such as DKA. Diabetes education needs to be repeated throughout the course of the illness and not just at diagnosis. In particular, parents need help on identifying the key features of DKA and how to distinguish it from other illnesses.
3. The adolescent years are the period when youth take on more responsibility for the management of their disease and become more independent, e.g., participating in events without their parents or guardians accompanying them. It is important that transition education begins during this time to ensure that they are able to identify issues with their health status and its complications [8, 24]. When a young child is diagnosed with diabetes, the parents receive most of the teaching; therefore re-education which is directed at the youth needs to occur as the child matures into adolescence and when transitioning into adult care. Peer-to-peer education maybe an effective way to educate youth.
4. When the youth is under the supervision of a teacher or other temporary caregiver, there is added burden, anxiety and responsibility on the family to ensure the caregiver is properly trained. There is a need for better resources to help parents, teachers and caregivers [4, 25]. Resources that may be beneficial to youth with diabetes are web-based videos, information sharing at diabetes camp, resources developed by youth themselves, or peer-mentoring by older adolescents or young adults. Parents need support when dealing with the education system.
5. Access to DKA prevention tool-kits and simple instructions on what to do may help lessen the stress of parents when addressing episodes of high blood sugars.

This study has a number of limitations, particularly around the recruitment of participants. For each focus group, there were eight confirmed participants before the focus groups, but participants ranged from 2 to 6. In the first urban focus group, five families had confirmed that they were going to attend, but only one family ultimately did, likely due to inclement weather on the evening of the focus group. Adult participants reported extra-curriculum activities made it difficult for youth to attend. One of the themes that came through the focus groups was participants’ dedication to the proper management of their condition. It is probably the case that the participants of the focus groups were already better-informed at managing their diabetes than the average diabetes population and may not have included youth at highest risk for DKA (e.g., youth dealing with issues of chronic family disruption or youth with behavior concerns). It is also likely that these patients have a closer connection to their diabetes care team and do not reflect high risks patients, such has those who deliberately do not adhere to their prescribed treatment regime.

## Conclusions

Based on the key findings arising from the focus groups, we have begun to develop some of the supports recommended by the participants. We developed and implemented an information campaign for all of the schools in NL, including resources for teachers and posters to schools to remind teachers of diabetes symptoms in youth. We are also in the process of developing videos about DKA in which youth with diabetes describe their experience. Finally, we have held educational sessions on DKA with families, primary care physicians and hospitals across the province on DKA prevention and we have established DKA treatment protocols to help medical staff more quickly identify undiagnosed cases of diabetes and properly manage DKA according to national and international guidelines. We are continuing to measure the collective impacts of these initiatives on DKA rates within the province.

## Appendix: Focus group questions

1. What is your understanding of the term diabetic ketoacidosis?
2. What is your understanding of the symptoms or signs of DKA?
3. What is your knowledge regarding the possible causes of DKA?
4. Think back to the time when your child was first diagnosed with diabetes. What information were you given about diabetic ketoacidosis?
5. Have you ever discussed with your child/teen about DKA?
6. Has a member of your diabetes health care team talked to you or your child/teen about DKA?
7. What has your family’s experience been with DKA?
8. How comfortable do you feel about managing sickness, such as “the stomach flu” at home with your child/teen with DKA?
9. In your opinion, what are some of the most significant barriers to proper insulin management and DKA prevention?
10. Diabetes clinics in other regions have developed resources such as websites to help patients and their families with DKA prevention. Are there any additional resources for Newfoundland and Labrador that would help you and your child with insulin management and DKA prevention?
11. Can you suggest an ideas or tools that would help families learn about and prevent DKA?

## Additional file

Additional file 1:
**Table S1.** Focus group locations, dates, participants, gender of youth participants, and cases of DKA discussed with cause.

## References

[1] Wolfsdorf J, Craig ME, Daneman D, Dunger D, Edge J, Lee W, Rosenbloom A, Sperling M, Hanas R (2009). Diabetic ketoacidosis in children and adolescents with diabetes. Pediatr Diabetes.

[2] Usher-Smith JA, Thompson MJ, Sharp SJ, Walter FM (2011). Factors associated with the presence of diabetic ketoacidosis at diagnosis of diabetes in children and young adults: a systematic review. BMJ.

[3] Rewers A, Klingensmith G, Davis C, Petitti DB, Pihoker C, Rodriguez B (2008). Presence of diabeteic ketoacidosis at diagnosis of diabetes mellitus in youth: the search for Diabetes in Youth Study. Pediatrics.

[4] Bismuth E, Laffel L (2007). Can we prevent diabetic ketoacidosis in children?. Pediatr Diabetes..

[5] Vanelli M, Chiari G, Ghizzoni L, Costi G, Giacalone T, Chiarelli F (1999). Effectiveness of a Prevention Program for Diabetic Ketoacidosis. Diabetes Care.

[6] King BR, Howard NJ, Verge CF, Jack MM, Govind N (2012). A diabetes awareness campaign prevents diabetic ketoacidosis in children at their initial presentation with type 1 diabetes. Pediatric Diabetes.

[7] Weber C, Kocher S, Neeser K, Joshi SR (2009). Prevention of diabetic ketoacidosis and self-monitoring of ketone bodies: an overview. Curr Med Res Opin.

[8] Rewers A (2012). Current Concepts and Controversies in Prevention and Treatment of Diabetic Ketoacidosis in Children. Curr DiabRep.

[9] Fleming E (2002). The transition of adolescents with diabetes from the children’s health care service into the adult health care service: a review of the literature. J Clin Nurs.

[10] Freeborn D, Dyches T, Roper SO, Mandleco B (2012). Identifying challenges of living with type 1 diabetes: child and youth perspectives. J of Clinical Nursing.

[11] Alaghehbandan R, Collins KD, Newhook LA, MacDonald D (2006). Childhood type 1 diabetes mellitus in Newfoundland and Labrador. Canada. Diabetes Res Clin Pract.

[12] Newhook LA, Curtis J, Hagerty D, Grant M, Paterson AD, Crummel C, Bridger T, Parfrey P (2004). High incidence of childhood type 1 diabetes in the Avalon Peninsula, Newfoundland. Canada. Diabetes Care.

[13] Newhook LA, Penney SJ, Fiander J, Dowden J (2012). Recent incidence of type 1 diabetes mellitus in children 0-14 years in Newfoundland and Labrador, Canada, climbs to over 45/100,000. BMC Research Notes.

[14] Rytkonen M, Moltchanova E, Ranta J, Taskinen O, Tuomilehto J, Karvonen M (2003). The incidence of type 1 diabetes among children in Finland–rural-urban difference. Health Place.

[15] Butcher MK, Gilman J, Meszaros JF, Bjorsness D, Madison M, McDowall JM, Oser CS, Johnson EA, Harwell TS, Helgerson SD, Gohdes D (2006). Improving access to quality diabetes education in a rural state: the Montana Quality Diabetes Education Initiative. Diabetes Educ.

[16] Reddy P, Hernan AL, Vanderwood KK, Arave D, Niebylski ML, Harwell TS, Dunbar JA (2011). Implementation of diabetes prevention programs in rural areas: montana and south-eastern Australia compared. Aust J Rural Health.

[17] Hale NL, Bennett KJ, Probst JC (2010). Diabetes care and outcomes: disparities across rural America. J Community Health.

[18] Cameron FJ, Clarke C, Hesketh K, White EL, Boyce DF, Dalton VL, Cross J, Brown M, Thies NH, Pallas G, Goss PW, Werther GA (2002). Regional and urban Victorian diabetic youth: clinical and quality-of-life outcomes. J Paediatr Child Health.

[19] The Rural Centre.: Rural and small town Canada analysis bulletin 3(3), November 2001. http://www.theruralcentre.com/doc/RST-Def_Rural.pdf.

[20] Green J, Thorogood N (2009). Qualitative methods for health research.

[21] NVivo 9:. QSR International. http://www.qsrinternational.com/products_nvivo.aspx.

[22] Health Research Ethics Authority Website. http://www.hrea.ca/Home.aspx.

[23] Sibley LM, Weiner JP (2011). An evaluation of access to health care services along the rural-urban continuum in Canada. BMC Health Serv Res.

[24] Buckloh LM (2008). Lochrie, Antal H, Milkes A, Canas JA, Hutchinson S, Wysocki T. Diabetes Complications in Youth. Diabetes Care.

[25] Marshall M, Gidman W, Callery P (2013). Supporting the acre of children with diabetes in school: a qualitative study of nurses in the UK. Diabet Med.

