# Supplementary material for: Reducing episodes of diabetic ketoacidosis within a youth population: a focus group study with patients and families
Source: BMC Res Notes. 2015 Sep 1;8:395. doi: 10.1186/s13104-015-1358-7 (PMC4553941; doi:10.1186/s13104-015-1358-7)
Supplement: Additional file 1: — Table S1. Focus group locations, dates, participants, gender of youth participants, and cases of DKA discussed with cause. [file 13104_2015_1358_MOESM1_ESM.docx]

**Tables**

**Table 1: Focus group locations, dates, participants, gender of youth participants, and cases of DKA discussed with cause.**

| **Focus Group Location** | **Date of Focus group** | **Number of Participants** | **Gender of Youth**  **Participants** | **Number of Cases of DKA discussed and cause identified** |
| --- | --- | --- | --- | --- |
| Urban 1 | November 22, 2011 | 2 (1 adult, 1 youth) | 1 Female | 2 cases for the same youth (other illness, cause unknown) |
| Rural 1 | December 7th, 2011 | 6 (5 adults, 1 youth) | 1 Male | None |
| Rural 2 | December 15th, 2011 | 5 (2 adults, 3 youth) | 2 Males; 1 Female | None |
| Urban 2 | March 6th, 2012 | 6 (all adults) |  | 3 (1 other illness, 2 new diagnosis) |
